# Parasitological, serological and molecular survey of *Trypanosoma evansi* infection in dromedary camels from Cholistan Desert, Pakistan

**DOI:** 10.1186/s13071-015-1002-3

**Published:** 2015-08-12

**Authors:** Sonia Tehseen, Nusrat Jahan, Muhammad Fiaz Qamar, Marc Desquesnes, Mirza Imran Shahzad, Stijn Deborggraeve, Philippe Büscher

**Affiliations:** Department of Zoology, Government College University Lahore, Lahore, Pakistan; CIRAD-Bios, UMR17 Inter Tryp, Montpellier, France; University College of Veterinary and Animal Sciences, Islamia University Bahawalpur, Bahawalpur, Pakistan; Department of Biomedical Sciences, Institute of Tropical Medicine, Antwerp, Belgium

**Keywords:** *Trypanosoma evansi*, Cholistan Desert, RoTat 1.2, ELISA/VSG RoTat 1.2, Immune trypanolysis, CATT/*T. evansi*, PCR, Pakistan

## Abstract

**Background:**

Surra, a vector borne disease caused by *Trypanosoma (T.) evansi,* affects the health, productivity and working capacity of camels. Since clinical signs are not pathognomonic, diagnosis must be confirmed by laboratory methods. This is a first study on the prevalence of surra in Cholistan Desert, Pakistan using a broad variety of diagnostic tests thereby emphasizing it as a risk for the dromedaries of Pakistan.

**Methods:**

In a cross sectional study, 1005 dromedary camels from three districts in the Cholistan Desert were sampled to assess the prevalence of trypanosomosis due to *T. evansi* by means of parasitological (Giemsa stained thin smear), serological (formol gel test, CATT/*T. evansi,* ELISA/VSG RoTat 1.2*,* immune trypanolysis) and molecular tests (TBR1/2 PCR and RoTat 1.2 PCR). Kappa was calculated to assess the degree of agreement between different tests whereas chi-square test along with odds ratios and their 95 % confidence intervals were used to study influence of breed, gender, age and locality on disease prevalence.

**Results:**

Overall prevalence was 0.7 % with Giemsa stained thin smears (GST), 40.1 % with formol gel test (FGT), 47.7 % with CATT/*T. evansi*, 44.2 % with ELISA/VSG RoTat 1.2, 39.9 % with immune trypanolysis (TL), 31.9 % with TBR1/2 PCR and 30.5 % with RoTat1.2 PCR. Based on these results, the Cholistan Desert appears to be a high risk area for surra. According to TL and TBR1/2 PCR, camels at Bahawalpur are approximately two times more likely to be infected than those in Bahawalnagar (OR = 1.8; 95 % CI: 1.38-2.42) and Rahim Yar Khan (OR = 1.9; 95 % CI: 1.30-2.75). Test agreement of TL was moderate with CATT/*T. evansi* (k = 0.43; 95 % CI: 0.378-0.489) and ELISA/VSG RoTat 1.2 (k = 0.54; 95 % CI: 0.489-0.594) and poor with the other tests. Test agreement between TBR1/2 PCR and RoTat1.2 PCR was almost perfect (k = 0.96; 95 % CI: 0.950-0.984). We didn't find evidence for the presence of *T. evansi* type B in the studied population.

**Conclusion:**

Our study supports using antibody detection tests, rather than parasitological and molecular examination, to assess surra prevalence in camels. It also calls for implementation of measures to control surra in the Cholistan Desert.

## Background

*Trypanosoma evansi* (*T. evansi*) was first isolated in 1880 from infected camels and equids in the Dera Ismail Khan district of the Punjab, in what today is Pakistan. It is a protozoan parasite of both intra- and extra vascular fluids of mammals causing the disease surra throughout tropical and subtropical areas of the world, including Asia, Africa and Latin America [1, 2]. It has a large diversity of mammalian hosts and has the ability to periodically switch its major variant surface glycoprotein (VSG), producing relapses of parasitaemia. These highly immunogenic VSGs determine the variable antigen type (VAT) of an individual trypanosome and induce the production of VAT specific protective antibodies with opsonising, agglutinating and lytic activity [3, 4]. The predominant VAT RoTat 1.2 is shown to be expressed in all *T. evansi* Type A isolates other than non RoTat 1.2 *T. evansi* type A and *T. evansi* type B that lack both RoTat 1.2 genes and their associated VSGs. Mostly, *T. evansi* isolates around the world are found to be genetically homogenous [5]. *T. evansi* is not cyclically transmitted; it is mechanically transmitted by hematophagous flies (*Tabanus*, *Atylotus*, *Chrysops*, *Haematopota*, *Lyperosia* and *Stomoxys*) and also biologically by vampire bats (*Desmondus rotundus*) in South America [6].

Camel trypanosomosis is a serious problem in camel husbandry and is a major threat to productivity and economic losses. Camels are also affected to a lesser extent by tsetse-transmitted trypanosome species like *T. simiae*, *T. brucei, T. congolense* and *T. vivax* [7]. The course of infection ranges from an acute form with high mortality to a chronic form, which is characterised by reduced fertility, generalised loss of body condition, neuropathy and immune suppression coupled with anaemia and eventually death in both domestic and wild mammals [8].

Absence of pathognomonic signs of the disease necessitates laboratory diagnosis to be carried out for confirmation of infection. Some routinely employed parasitological tests in ascending order of sensitivity are microscopic examination of fresh or stained blood smears, microhaematocrit centrifugation technique (MHCT), miniature anion-exchange centrifugation technique (mAECT) and rodent inoculation. Antigen based tests might complement parasitological tests to detect active infection, but they suffer from low sensitivity owing to fluctuating parasitaemia, low parasite numbers in chronic infections and presence of antigen-antibody complexes. Various studies have used an antigen-detecting latex agglutination test (Suratex) and an antigen ELISA to asses prevalence of surra in camels [9–13].

As an alternative to parasitological tests, serological immunoassays can be applied for diagnosis of surra. Non-specific antibody tests like the formol gel, the mercuric chloride and the thymol turbidity test are routinely used for screening surra in poor-resource laboratories. More sophisticated antibody detection tests such as the card agglutination test (CATT/*T. evansi*) [14], the immune trypanolysis (TL) assay [3], the latex agglutination test [15], the immunofluorescence antibody test (IFAT) [16] and the enzyme-linked immunosorbent assay for *T. evansi* have been employed in different studies on camel trypanosomosis [17–21]. Although sensitive and accurate, these tests cannot differentiate between past and recent infections.

Polymerase chain reaction (PCR) is considered to be superior to parasite detection and antigen detection tests due to its sensitivity in detecting the prepatent and chronic phase of an infection. PCR is reported to be able to detect 1 trypanosome/ml of blood or as low as 1 pg of *Trypanosoma* DNA in the presence of host DNA [22, 23]. For molecular analysis, various target sequences such as kinetoplast DNA, ribosomal DNA, internal transcribed spacer region and VSG genes are reliable targets for the detection of *T. evansi* [24].

After the outbreak of surra in camels during 1985–1986 in Baluchistan, several studies on the prevalence of surra in camels have been conducted in different areas of Pakistan [1, 25–28]. According to the Food and Agriculture Organization Statistics (FAOSTATS), Pakistan is supporting approximately 1.0 million head of dromedary camels [29]. The impact of the disease on camels in Pakistan and subsequent economic loss is currently difficult to assess because of lack of diagnostic tools for judging the extent of its incidence, prevalence and morbidity in the field. The aim of the study was to assess the prevalence of camel trypanosomosis in the Cholistan Desert and to compare the diagnostic effectiveness of several parasitological, serological and molecular diagnostics.

## Methods

### Study site

**Fig. 1:**
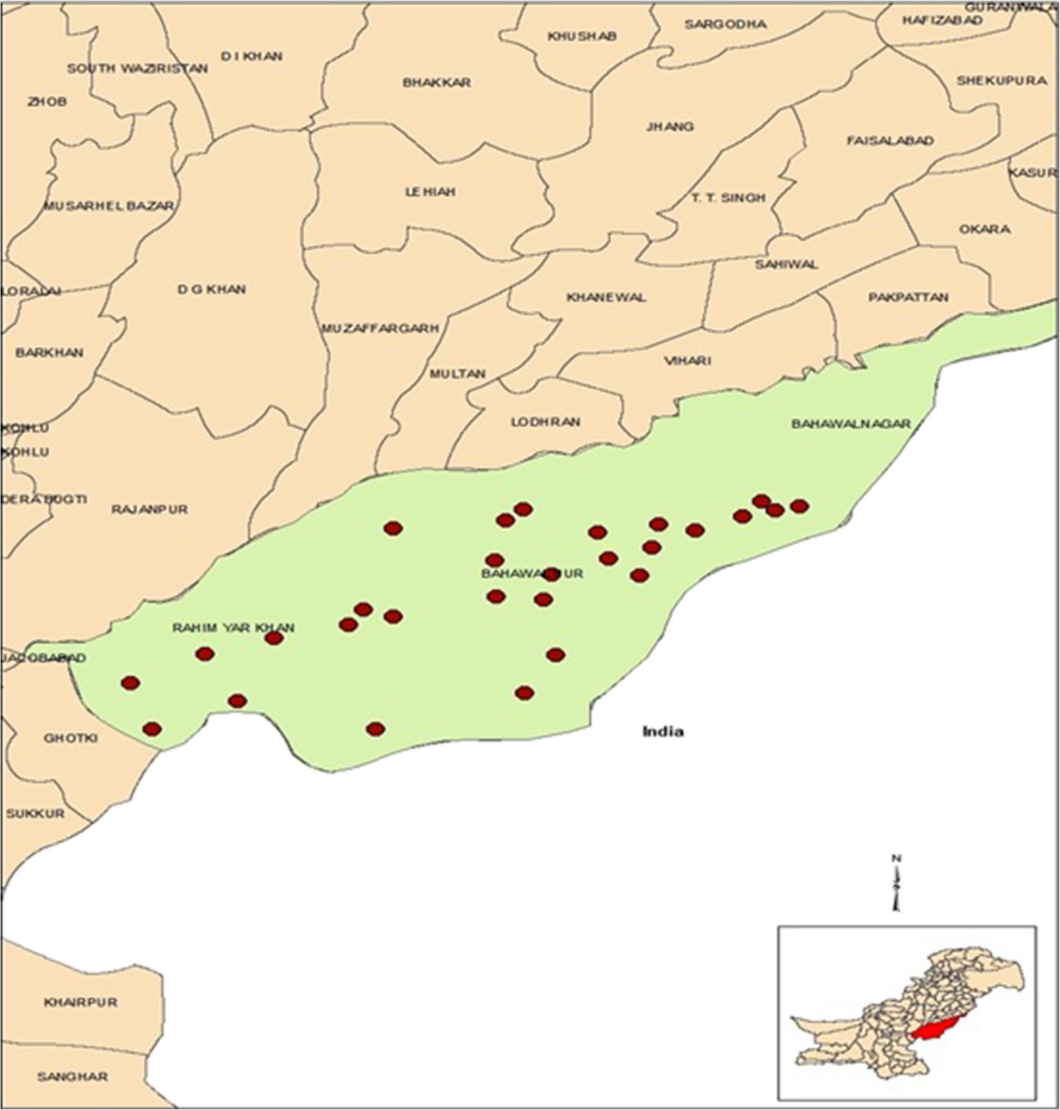
Map of Pakistan with the Cholistan Desert and the three districts. Localisation of sampling sites is shown in red dots

The hot and hyper arid sandy Cholistan Desert is an extension of the Great Indian Desert and stretches over an area of 26,330 Km^2^ (Fig. 1). It lies in the Southern Punjab of Pakistan between latitudes of 27° 42’ and 29°45’N and longitudes of 69° 52’ to 75° 24’E and comprises three districts, Rahim Yar Khan, Bahawalpur and Bahawalnagar. The Cholistan Desert is classified as a “tropical desert” with annual mean temperatures lying between 20 °C and 40 °C. The mean annual rainfall varies from less than 100 mm in the west to 200 mm in the east. Absence of permanent water bodies is compensated by collection of rainwater in artificial ponds known as “*tobas*” in local dialect. Drying up of *tobas* causes migration of nomads and animals to semi-permanent settlements where primitive unlined wells and *kunds* (usually lined) are available and drying up of the latter causes migrations to peripheral areas of the desert where water and fodder are available [30].

The total livestock population of the Cholistan Desert is estimated to be 1.56 million with camels (0.08 million head), cattle (0.67 million), sheep (0.45 million) and goats (0.35 million) as the predominant types of livestock. Insufficient water, nutrition, non-domesticated production system, marketing, animal health and production services are some of the constraints for livestock productions [31].

The vegetation coverage is poor. *Calligonum polygonoides, Acacia jacquemontii, Haloxylon recurvum*, *Haloxylon salicornicum, Salsola baryosma*, *Suaeda fruticosa, Capparis decidua, Prosopis cineraria*, *Panicum turgidum*, *Sporobolus iocladus, Aeluropus lagopoides, Crotalaria burhia*, *Aerva persica, Calotropis procera, Pulicaria rajputanae* serve as a common source of fodder for camels [32]. Hematophagous flies such as *Tabanus spp*, *Hematobia spp*. and *Stomoxys spp* are abundant (unpublished data).

### Study animals

The study population consisted of dromedary camels of all age groups residing in Cholistan and managed under pastoral production systems. Within the three districts of Cholistan Desert, specific study sites were chosen purposively where camels congregated for watering and browsing purposes. Every fifth animal (n = 1005) was selected from the population at different grazing and watering points on a monthly basis from January 2012 to December 2013. The age of the animals recorded was based on information from the owners. Camels below 2 years of age were considered as calves, those between 2 to 4 years as young animals, while those above 4 years of age were considered as adults. Each animal was examined clinically and information on different aspects like age, gender, breed, month of sampling, previous trypanocide treatment, history of abortions and weight estimations were also recorded [33].

### Blood sampling

Ten ml of blood were collected from the jugular vein. Four ml of blood were placed into a tube containing EDTA for packed cell volume (PCV) measurement and for DNA extraction and was stored in cool boxes until transported to the laboratory. It was later aliquoted into labelled microtubes and preserved at −80 °C till further analysis. For serological tests, 3 ml of blood were placed in serum separator tubes with clot activators. After centrifugation for 15 min at 2000 rpm, serum was aliquoted and stored at −80 °C. Serum volumes of 200 μl were later spotted onto Whatman filter paper No. 4 for further analysis.

### Ethical approval

The study protocol was conducted with the ethical approval of Advance Studies And Research Board (ASRB) of the Government College University, Pakistan. The protocol for the culture of trypanosomes in mice was approved by the Veterinary Ethical Committee of the Institute of Tropical Medicine Antwerp, Belgium (ITM) (BM2013-7).

### Parasitological examination

A small drop of blood (3–5 μl) was placed at one end of a clean microscope slide and a thin film was drawn out. It was air-dried and fixed in methyl alcohol for 1 min and allowed to dry. The smears were stained with Giemsa (one drop of Giemsa + 1 ml PBS, pH 7.2) for 25 min, followed by washing of the slides in tap water and drying. The Giemsa stained thin smears (GST) were examined at a magnification of 400–1000x with oil immersion [34].

### Determination of packed cell volume (PCV)

Approximately 70 μl of blood were put into a heparinised capillary tube (75 × 1.5 mm). The dry end was closed with plasticine and centrifuged at 14000 rpm for 5 min. The PCV was expressed as percentage of the total blood volume [34].

### Serological analysis

The formol gel test (FGT) was carried out by adding two drops of concentrated formalin solution (37 % formaldehyde) to 1 ml of serum. The test was considered positive if the serum coagulated immediately and turned white [34].

Immune trypanolysis (TL) was carried out according to the protocol of Holland *et al*. [35] with *T. evansi* VAT RoTat 1.2. One confetti of 6 mm diameter was punched out from each filter paper having sera blotted onto it and later transferred to a flat bottom polystyrene microtitre plate (Greiner Bio-One, Wemmel, Belgium). Twenty μl of guinea pig serum were poured onto each filter paper and allowed to be absorbed for 5 min. A suspension of 5–10 trypanosomes per microscopic field (40 x magnifications) was made in guinea pig serum and 10 μl of this trypanosome suspension were added to each sample well and the plate was shaken for 90 min. One drop of the trypanosome suspension was examined under the microscope and the percentage of lysed trypanosomes was recorded. If 50 % or more of the trypanosomes were lysed, the test was considered positive.

ELISA/VSG RoTat 1.2 was carried out as described by Verloo *et al*. [18]. For coating ELISA plates, the purified antigen (VSG RoTat 1.2) was diluted to a concentration of 2 μg/ml in PBS buffer (0.01 M; pH 7.4; NaCl 8.2 g/l; NaH_2_PO_4_.H_2_O 0.2 g/l; Na_2_HPO_4_.2H_2_O 1.44 g/l). One hundred fifty μl of the diluted antigen were added per well and stored at −4 °C overnight. Half of the wells were left empty as antigen negative control. The antigen was removed and 350 μl of PBS-Blotto (0.01 M; pH 7.4; NaCl 11.7 g/l; NaH_2_PO_4_.H_2_O 0.2 g/l; Na_2_HPO_4_.2H_2_O 1.44 g/l; NaN_3_ 0.5 g/l; skimmed milk powder 10 g/l) were added per well for blocking the plate. The plate was then incubated for one hour at room temperature followed by three times washing with a 1 min interval using 350 μl of PBS-Tween (PBS with Tween 20 0.5 ml/l) per well. Sera collected on filter paper were eluted by punching out one confetti of 6 mm diameter from each sample and adding 1.0 ml of PBS Blotto-Tween for overnight incubation at 4 °C. A volume of 150 μl of the eluted fraction was added in duplicate to an antigen coated and an antigen free well in an ELISA plate and incubated at room temperature for an hour. Washing was done three times with 350 μl of PBS-Tween prior to addition of 150 μl/well of protein A peroxidase conjugate (1/10,000 dilution in PBS-Tween). Plates were incubated for one hour at room temperature followed by five times washing with 350 μl of PBS-Tween where after 150 μl of substrate-chromogen solution were added per well and incubated for 60 min at room temperature. Absorption was read at 414 nm in a Labsystems Multiskan RC ELISA reader. The corrected optical density (O.D.) of each sample and of the serum controls was calculated by subtracting the mean O.D. of the two antigen negative wells from the mean O.D. of the two corresponding antigen containing wells. These corrected O.D.s were expressed as percentage of the O.D. obtained with the strong positive control included in each plate (percent positivity, P.P.). If for a given sample the difference between the two raw O.D.s was more than 25 % of their mean, the results were rejected and the sample retested. Cut-off value for ELISA–confetti was calculated to be 14 %.

Sera were also tested for the presence of anti-*T. evansi* antibodies using the card agglutination test for *T. evansi* (CATT/*T. evansi*) (Institute of Tropical Medicine, Antwerp, Belgium). Approximately, 45 μl of the antigen were transferred onto the test card and mixed with 25 μl of the test sera diluted at 1/4 with PBS pH 7.2 as per manufacturer’s instructions. The card was agitated for 5 min and the reaction was checked in the clear light. Positive reaction was confirmed on recording agglutinations (blue agglutinates) [14].

### Molecular analysis

Three molecular tests were performed which are able to detect genomic DNA of *T. evansi* and therefore are not affected by the existence of dyskinetoplast *T. evansi* strains [36, 37]. The TBR1/2 PCR amplifies mini-chromosome satellite repetitive sequences and is considered the gold standard for detection of *Trypanozoon* DNA and allows as little as 1–5 trypanosomes/ml of blood to be detected [34, 38]. The RoTat 1.2 PCR is specific for *T. evansi* type A and is capable to detect as few as 50 trypanosomes/ml of blood [39]. *T. evansi* type B PCR targets minicircle sequences [39].

Genomic DNA was extracted from 250 μl of whole camel blood using a commercially available kit (Pure link, Genomic DNA minikit, Invitrogen) and was stored at −80 °C till further use.

One pair of *Trypanozoon-*specific and two pairs of species-specific primers were employed for analysis (Table 1).

**Table 1:** Details on the primers used in the molecular tests

Name	Specificity	Target	Primersequence	Reference
TBR1/2	*Trypanozoon*	Minichromosome satellite repetitive sequence	TBR1-F:5’GAATATTAAACAATGCGCAG 3’	[38]
			TBR2-R:5’CCATTTATTAGCTTTGTTGC 3’	
RoTat 1.2	*Trypanosoma evansi* type A	RoTat 1.2 VSG	RoTat-F:5’-GCGGGGTGTTTAAAGCAATA-3	[39]
			RoTat-R:5’-ATTAGTGCTGCGTGTGTTCG-3	
Eva B	*Trypanosoma evansi* type B	Minicircle genes	EVAB1-5’CACAGTCCGAGAGATAGAG-3	[57]
			EVAB2-5’CTGTACTCTACATCTACCTC-3	

TBR1/2 PCR was carried out in a 25 μl reaction mixture containing 2x gold Taq Master Mix (Invitrogen), 0.8 μM forward and reverse primers and 2.5 μl of template DNA. The cycles included activation at 95 °C for 5 min, followed by 30 cycles of denaturing at 95 °C for 30 s, annealing at 55 °C for 30 s and extension at 72 °C for 30 s. Final elongation was continued at 72 °C for 5 min.

RoTat 1.2 PCR was carried out in a 25 μl reaction mixture containing1x Hot Star Taq Master Mix (Qiagen), 0.8 μM of forward and reverse primers and 1.0 μl of template DNA. The cycles included activation of the hot start *Taq* polymerase at 94 °C for 15 min, followed by 40 cycles of denaturation at 94 °C for 30 s, primer annealing at 52 °C for 30 s, and elongation at 72 °C for 30 s. Final elongation was continued at 72 °C for 5 min.

EVAB PCR was carried out in 25 μl reaction mixtures containing 1x Hot Star Taq Master Mix (Qiagen), 0.8 μM of each primer and 1.0 μl of template DNA. The cycling conditions for PCR were an initial denaturation step at 94 °C for 15 min, followed by 30 cycles of denaturing at 94 °C for 30 s, annealing at 60 °C for 30 s and extension at 72 °C for 60 s. A final elongation was continued at 72 °C for 10 min. A positive (reference *T. evansi* DNA, 10 ng/ml) and a negative control (sterile water) were included for each PCR reaction. Amplified products were analysed by electrophoresis in a 2 % agarose gel (Eurogentec, Belgium) and UV illumination (Syngene, UK) after ethidium bromide staining of the DNA (Sigma, Belgium).

### Statistical analysis

Statistical analysis was performed using SPSS version 15. Levels of agreement between diagnostic tests were indicated by kappa values (k) and interpreted according to Landis and Koch [40] and the influence on prevalence of various factors, such as breed, gender, age and locality was determined using chi-square test along with odds ratios and their 95 % confidence intervals.

## Results

### Degree of agreement between the different diagnostic tests

Cross tables and degree of agreement for all test combinations are represented in Table 2.

**Table 2:** Cross-tables and degree of agreement between the different diagnostic tests. TL = immune trypanolysis, GST = Giemsa stained thin smear, FGT = formol gel test, TL = immune trypanolysis, CATT = CATT/*T. evansi*, ELISA = ELISA/VSG RoTat 1.2, pos = positive, neg = negative, CI = 95 % confidence interval, k = kappa with the following interpretation of agreement: <0 = poor, 0–0.2 = slight, 0.21-0.4 = fair, 0.41-0.6 = moderate, 0.61-0.8 = substantial, 0.81-1 = almost perfect

		FGT		CATT		ELISA		TL		TBR1/2		RoTat 1.2	
		pos	Neg	pos	neg	pos	neg	pos	neg	pos	neg	pos	neg
GST	pos	7	0	7	0	1	6	7	0	7	0	7	0
	neg	396	602	472	526	443	555	394	604	314	684	300	698
	k (95 % CI)	0.02 (0–0.096)		0.02 (0–0.080)		<0		0.02 (0–0.096)		0.03 (0–0.119)		0.03 (0–0.123)	
FGT	pos			222	181	164	239	192	211	114	289	113	290
	neg			257	345	280	322	209	393	207	395	194	408
	k (95 % CI)			0.12 (0.058-0.182)		<0		0.13 (0.066-0.193)		<0		<0	
CATT	pos					301	178	299	180	157	322	152	327
	neg					143	383	102	424	164	362	155	371
	k (95 % CI)					0.36 (0.300-0.416)		0.43 (0.378-0.490)		0.02 (0–0.079)		0.02 (0–0.086)	
ELISA	pos							310	134	142	302	137	307
	neg							91	470	179	382	170	391
	k (95 % CI)							0.54 (0.489-0.594)		0.00 (0–0.065)		0.01 (0–0.070)	
TL	pos									136	265	131	270
	neg									185	419	176	428
	k (95 % CI)									0.04 (0–0.100)		0.04 (0–0.103)	
TBR1/2	pos											307	14
	neg											0	684
	k (95 % CI)											0.97 (0.951-0.985)	

Considering that molecular tests are surrogates for parasite detection, it is clear that agreement between parasite detection with GST and DNA detection with TBR1/2 PCR (k = 0.03) and RoTat 1.2 PCR (k = 0.03) is slight, as it is with all serological tests (k = 0.02) except ELISA/VSG RoTat 1.2with which agreement is poor (k < 0). On the other hand, agreement between the two DNA detection tests, TBR1/2 PCR and RoTat 1.2 PCR, is almost perfect (k = 0.97). Agreement between the non-specific FGT and the other serological tests varies from poor to slight (k ≤ 0–0.2) while agreements between all tests that specifically detect antibodies against RoTat 1.2 VSG range from fair to moderate (k >0.21 - 0.6) (CATT/*T.evansi* and ELISA/VSG RoTat 1.2: k = 0.36; CATT/*T. evansi* and TL: k = 0.43; ELISA/VSG RoTat 1.2 and TL: k = 0.54). Agreement between all serological tests and the molecular tests is poor to slight (k ranging from < 0 to 0.04).

### Overall prevalence

Using different tests, the overall prevalence estimates were statistically different from each other (*p* < 0.0005). Among1005 camels screened for *T. evansi* infection, the overall prevalence was found to be 0.7 % (95 % CI: 0.08-1.32) with GTS, 40.1 % (95 % CI: 37.07-43.13) with FGT, 47.7 % (95 % CI: 44.57-50.75) with CATT/*T.evansi*, 44.2 % (95 % CI: 41.11-47.25) with ELISA/VSG RoTat 1.2*,* 39.9 % (95 % CI: 36.87-42.93) with TL, 31.9 % (95 % CI: 29.06-34.82) with TBR1/2 and 30.5 % (95 % CI: 27.70-33.40) with RoTat1.2 PCR (Table 3).

**Table 3:** Number and percentage (%) of positive samples in the different diagnostic tests. Data are arranged according to district, breed, gender and age group. GST = Giemsa stained thin smear, FGT = formol gel test, TL = immune trypanolysis, CATT = CATT/*T. evansi*, ELISA = ELISA/VSG RoTat 1.2, pos = positive, neg = negative

			GST	FGT	CATT	ELISA	TL	TBR1/2	RoTat1.2
		Total	pos (%)	pos (%)	pos (%)	Pos (%)	pos (%)	pos (%)	pos (%)
District	Bahawalpur	420	7 (1.1)	259 (61.7)	224 (53.3)	210 (50.0)	205 (48.8)	95 (22.6)	94 (22.4)
	Bahawalnagar	399	0 (0.0)	144 (36.1)	202 (50.6)	156 (39.1)	13 7(34.3)	155(38.9)	152 (38.1)
	Rahim Yar Khan	186	0 (0.0)	0 (0.0)	53 (28.5)	78 (41.9)	59 (31.7)	68 (36.6)	58 (31.2)
Breed	Brella	420	3 (0.7)	245 (58.3)	201 (47.9)	171 (40.7)	166 (39.5)	119 (28.30)	118 (28.1)
	Mareecha	585	4 (0.7)	158 (27.0)	307 (52.5)	273 (46.7)	235 (40.2)	199 (34.0)	186 (31.8)
Gender	Male	440	1 (0.2)	105 (23.9)	196 (44.5)	200 (45.5)	153 (34.8)	164 (37.3)	155 (35.2)
	Female	565	6 (1.1)	298 (52.7)	283 (50.1)	244 (43.2)	248 (43.9)	154 (27.3)	149 (26.4)
Age	Adult	659	7 (1.1)	302 (45.8)	328 (49.8)	295 (44.8)	267 (40.5)	214 (32.5)	208 (31.6)
	Young	187	0 (0.0)	64 (34.0)	90 (48.1)	82 (43.9)	70 (37.4)	53 (28.3)	49 (26.2)
	Calf	159	0 (0.0)	37 (23.3)	61 (36.4)	67 (42.1)	64 (40.1)	51 (32.1)	47 (29.6)
Total		1005	7 (0.7)	403 (40.1)	479 (47.7)	444 (44.2)	401 (39.9)	321 (31.9)	307 (30.5)

### Prevalence according to district

All parasitological positive cases were reported from the Bahawalpur (Table 3). Using TL as gold standard test for *T. evansi* specific antibodies, the highest seroprevalence was reported from Bahawalpur 48.8 % (95 % CI: 37.1-43.1), followed by Bahawalnagar 34.3 % (95 % CI: 37.1-43.1) and 31.7 (95 % CI: 25.0-41.03). The seroprevalence estimates from Bahawalpur and Rahim Yar Khan were found to be significantly different (χ^2^ = 24.2, df = 1, *p* = 0.0001). Molecular prevalence estimates using both TBR1/2 PCR and RoTat 1.2 PCR were found to be significantly lower at Bahawalpur than both Rahim Yar Khan (χ^2^ = 11.34, df = 1, *p* = 0.001, χ^2^ = 4.43, d =1, *p* = 0.035) and Bahawalnagar (χ^2^ = 23.0.7, df = 1, *p* = 0.000, χ^2^ = 21.76, df = 1, *p* = 0.0005). Applying TL, and TBR1/2, the camels at Bahawalpur are approximately two times more likely to be infected than those in Bahawalnagar (OR = 1.8; 95 % CI: 1.38-2.42) and Rahim Yar Khan (OR = 1.89; 95 % CI: 1.30-2.75) respectively.

### Prevalence according to gender

Six out of the seven parasitologically positives were females (Table 3). Using TL as gold standard test for antibodies, females were found to have a significantly higher prevalence than males (χ^2^ = 8.6, df = 1, *p* = 0.0034). On the contrary, a higher molecular prevalence was observed in males than in females (χ^2^ = 9.9, df = 1, *p* = 0.002). Molecular estimates by both TBR1/2 PCR and RoTat1.2 PCR, show that males were 1.5 times more likely to be infected than females (OR = 1.57; 95 % CI: 1.2-2.1, 1.56; 95 % CI: 1.15-1.97).

### Prevalence according to breed

No significant differences were found between prevalence in breeds with any diagnostic test other than FGT (χ^2^ = 99.9, df = 1, *p* = 0.000) (Table 3).

### Prevalence according to age group

All GST positives were adults resulting in a significant difference in prevalence estimates between adults on the one hand and young and calves on the other hand (χ^2^ = 9.9, df = 1, *p* = 0.002) (Table 3). None of the other applied diagnostic tests showed a significantly different prevalence between these two groups.

### Packed cell volume

**Fig. 2:**
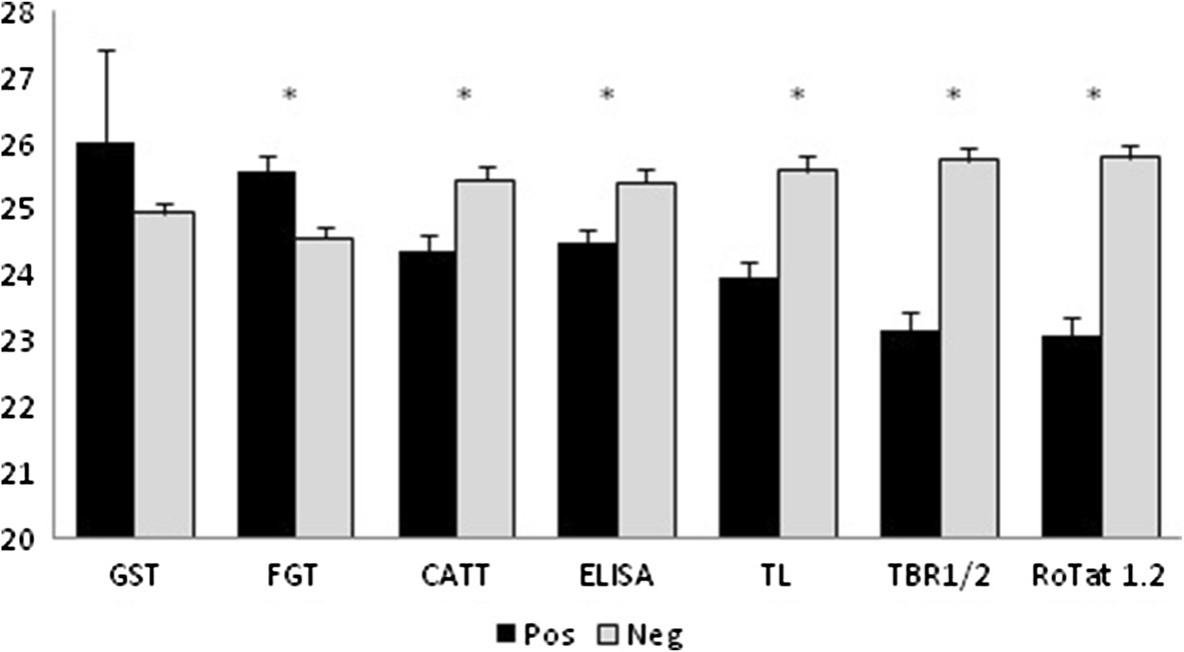
Mean PCV with standard deviation according to status in the different diagnostic tests. GST = Giemsa stained thin smear, FGT = formol gel test, TL = immune trypanolysis, CATT = CATT/*T. evansi*, ELISA = ELISA/VSG RoTat 1.2, pos = positive, neg = negative. * = significant difference between positives and negatives (*p* < 0.05)

PCV in function of status in the different diagnostic tests is represented in Fig. 2. Mean PCVs were non significantly and significantly higher for both parasitologically positive and FGT positive animals respectively (*p* < 0.05). On the other hand, animals that were positive in all the other serological tests and the molecular tests had a significantly lower average PCV than negative animals. Significant differences in PCV were also observed between PCR positive and negative animals in all the groups (age, breed, gender, and districts).

## Discussion

In this study, we aimed at investigating the prevalence of camel trypanosomosis in the Cholistan Desert, assessed by different diagnostic techniques including parasite detection, serology and molecular diagnostics.

Parasitological prevalence, assessed by Giemsa stained thin smear was much lower (0.07 %) than molecular prevalence (30.5 - 31.9 %), which is not unexpected given the high sensitivities of molecular tests. More animals could have been confirmed with trypanosomes if parasitological examination was performed with the haematocrit centrifugation technique (HCT) or by mouse inoculation but for this study, we had no access to both techniques in the field. If we consider the molecular tests as 100 % specific, the very high ratio of PCR- over GST-positives can be explained by low parasitaemia levels typical for the chronic stage of infection [41]. With about 1/3 of the camels actually infected, *T. evansi* infection is highly prevalent in the study population and definitely higher than what was observed in other studies in camel and in buffaloes in Pakistan [26, 27, 41]. This high prevalence rate is also reflected in the serological results where about 40 % of the study population showed detectable antibodies against *T. evansi* RoTat 1.2 in the immune trypanolysis, the reference antibody test which is considered 100 % specific [15]. Since antibodies can remain in the circulation for several months after cure and since only 17 % of all animals had no history of anti-trypanosome treatment (data not shown), it is not surprising that serological prevalence is higher than molecular and parasitological prevalence. The high proportion of positive specimens recorded with the different tests proves that surra is highly prevalent in the Cholistan Desert, similar to other regions, e.g. in Kenya [12, 33]. Since this is the first time that CATT/*T. evansi* and ELISA/VSG RoTat 1.2 are used in Pakistan, we cannot compare our data with those from other seroprevalence studies on surra in Pakistan except the study of Nadeem et al. who found 6 % of 200 horses from Gujranwala district positive in an immunofluorescence test [42]. One other study by Aslam et al. reported 21.6 % prevalence in 120 equine samples using antibody ELISA kit (FAO/IAEA, Vienna, Austria) [43].

Although the agreement between GST and the other diagnostic tests was poor, all parasitologically positive animals were also positive in FGT, TBR1/2 PCR and the different RoTat 1.2-based tests except for one animal that was negative in ELISA/VSG RoTat 1.2. This finding is consistent with the earlier studies of Verloo et al. where it was shown that the majority of *T. evansi* type A strains from all over the world express the RoTat 1.2 VSG shortly after infection [44]. Only one study described the existence of *T. evansi* type A strains from Kenya that doesn't contain the RoTat 1.2 VSG gene [5]. The fact that one parasitologically confirmed animal was negative in ELISA/VSG RoTat 1.2 but positive in TL, can be explained by the use of filter paper eluate instead of plasma, thus reducing the sensitivity of ELISA and/or by the higher analytical sensitivity of TL [35].

Except for the fact that all parasitologically confirmed animals were positive in the FGT, this serological test showed poor to slight accordance with any other diagnostic test for surra applied in the present study. The high numbers of discordant specimens confirms the poor sensitivity and poor specificity of the FGT compared to the other diagnostic tests reported in many other studies, including on visceral leishmaniasis in human [45]. The apparently poor specificity of the FGT is explained by the fact that it detects, in a non-specific way, high concentrations of plasma immunoglobulins that can be due to any acute or chronic infection causing hypergammaglobulinemia. The fair to moderate agreement among the other, VSG specific, serological tests is expected from what has been observed in comparative studies on experimentally and in naturally infected animals [15, 18, 35, 44]. Among the antibody detection tests for surra as well as for human African trypanosomosis, the TL is considered to be highly, if not 100 % specific [3, 35, 46]. Thus, with TL as gold standard for antibody detection, false positives in CATT/*T. evansi* and ELISA/VSG RoTat 1.2 are explained by the exposure of cross-reacting epitopes in the CATT and ELISA antigens while false negatives may be the result of the higher analytical sensitivity of TL [35].

Concordance of CATT/*T. evansi*, ELISA/VSG RoTat 1.2 and TL with the molecular tests is poor. False positives can be due to specific antibodies that remain present after treatment as explained above. On the other hand, the high number of false negatives in serology compared to the molecular tests is puzzling and cannot be explained by the use of filter paper eluates for ELISA/VSG RoTat 1.2 and TL since a similar number of false negatives is also observed in CATT/*T. evansi* that was performed on plasma. The almost perfect concordance between the two molecular tests targeting totally different sequences in the trypanosome genome strongly indicates the presence of trypanosome DNA in these serologically negative specimens and we didn't find any positive reaction in the *T. evansi* type B specific EVAB PCR (data not shown). In addition, from the negative controls included during the DNA extraction and in the PCR, we couldn't find evidence of cross-contamination. Therefore, the serologically false negatives must remain unexplained unless we consider the molecular tests not as fully specific or we accept that between 15 % and 18 % of all studied animals carried recent infections and were sampled prior to the appearance of RoTat 1.2 specific antibodies.

In this study, a high prevalence of surra has been observed in all three districts of Cholistan Desert using different techniques. In addition to finding all GTS positives from Bahawalpur, highest seroprevalence is also reported from the same district. On the contrary, the molecular prevalence is reported to be highest at Bahawalnagar thereby suggesting that Cholistan is a high risk area of surra affected from both past and recent active infection. This can be explained by ecological similarity and presence of infrastructure in terms of connectivity between different districts and equally distributed heavy spells of rain as experienced lately. This is directly related to vector abundance and a higher dissemination rate of the disease [8].

The fact that there was a significantly higher seroprevalence in females and a higher molecular prevalence amongst males shows that both sexes are highly susceptible to disease although males were having a more active infection at time of sampling. Moreover, six of the seven parasitologically positive animals were females. The higher prevalence in adult females might be due to pregnancy and lactation, which may reduce resistance in female camels and render them more susceptible to infection [25]. On the other hand, increased infection in males can be explained by the fact that male camels may be in stress due to fatigue owing to physical work, travelling in terms of searching for food and water, and hence more exposure to vectors. Bogale et al. reported a greater prevalence in males than females while studies by Ngaira et al. reported no difference in prevalence [47, 48]. Mareecha camels were reported to have a greater prevalence than Brella although the difference was not significant.

In the current studies, higher prevalence estimates in adult population than non-adult camels is in agreement with the studies of Jacquiet et al. and Diall et al. [49, 50]. This can be attributed to various factors like overestimation of disease owing to, persistence of antibodies following treatment, chronic nature of infection and intermittent parasitaemia, stress, poor management, draught, poor silage and preference by vectors because of larger surface area. Non-adults are often reported to be less affected group as they may go unnoticed because of high mortality owing to severity of disease, preference by the pastoralists to keep these animals in the same residing area and restricted movement to distant vector abundant areas [51–53]. In the non-adult population, TL and both PCRs gave higher prevalence in calves than young camels unlike CATT and FGT. Hence it can be concluded that although young camels are more susceptibility to surra after waning of maternally transferred immunity, a higher prevalence in calves can be attributed to the fact that both the groups were reared in the same area and were exposed to the same risk [54].

Anemia is considered as one of the characteristic findings in animals suffering from surra [55]. Therefore; the finding of higher PCV values in parasitologically positive animals is unexpected but may be attributed to the fact that GST detected only a small fraction of actually infected animals rendering the difference in PCV between parasitologically positive and negative animals non-significant. The significantly higher PCV in FGT positive animals is also unexpected but may be explained by our data proving that FGT is not accurately detecting active infection with *T. evansi,* which is further illustrated by the poor agreement of FGT with any other diagnostic test used in this study. On the other hand, animals that were positive in the serological tests had a significantly lower PCV. This lower PCV is even more obvious in the animals with a positive result in the molecular tests, thus strengthening the suggestion that molecular tests are more accurate in detecting active infection than serological tests since the latter may remain positive after cure. In various studies on surra in camels, PCV cut-off values have been proposed for diagnostic purposes such as 23 %, 20 % and 18 % [47, 50, 56]. In principle, our data would also allow to propose a cut-off value (e.g. 23.5 %) but it is questionable that a difference of 2 % between infected and non-infected animals is sufficient to accurately diagnose an infection. This does not exclude that the combination of a low PCV and positivity in a molecular and, especially, in a serological test may have a diagnostic value.

Our study has some limitations. First of all, we were not able to isolate *T. evansi* strains. Some mice inoculated with the blood of parasitologically confirmed camels became parasitaemic within a few days but died before a cryostabilate could be prepared. Secondly, no rigorous and complete clinical data collection was performed. We can only report that 3 out of the 7 parasitologically confirmed animals showed signs of acute infection with fever, anorexia, generalised oedema and eventually died, whereas the 4 other showed signs of chronic infection such as progressive loss of body weight, intermittent high fever, muscular atrophy, pale mucous membrane, blindness, characteristic sweet odour due to urinary ketones and faulty gait. These chronic cases were successfully treated with Trypamidium (0.5 mg/kg).

## Conclusion

According to our knowledge, this is the first report on *T. evansi* infection in camels in Pakistan making use of a combination of parasitological, serological and molecular tests, including those that are recommended by OIE. This study indicates that surra caused by *T. evansi* type A is circulating in dromedary camels of all age groups, breeds and gender in all districts of the Cholistan Desert. Since no test is 100 % sensitive and 100 % specific and not all tests can be applied in the field, the choice of which test to deploy during surveys and in routine diagnostic practice will depend on the test characteristics. For routine diagnostic practice, the CATT/*T. evansi* is appropriate but decision to treat will not only depend on CATT positivity but also on the clinical aspect and history of trypanocidal treatment. In case parasitological examination is desired, GST has no value and should be replaced by concentration techniques like MHCT or mAECT. For large scale surveys that can rely on proper laboratory facilities, ELISA/VSG RoTat 1.2 is more appropriate and cheaper than PCR and can be performed on filter paper eluates. Given the high prevalences observed, we recommend to establish measures to decrease disease incidence by treatment of infected animals and by controlling the vector. We further recommend to investigate the prevalence of *T. evansi* in other domestic animals, like sheep and goats, living in the same environment. Finally, we recommend to isolate the *T. evansi* strains circulating in the Cholistan Desert for extended studies on their pathogenicity, genetic relationship with strains from other geographical origin and drug sensitivity.

## Abbreviations

CATT: Card agglutination test for trypanosomosis
FGT: Formol gel test
GST: Giemsa stained thin smear
RoTat: Rhode *Trypanozoon* antigenic type
TL: Immune trypanolysis
VSG: Variant surface glycoprotein
